# ACCESS: an empirically-based framework developed by the International Nursing CASCADE Consortium to address genomic disparities through the nursing workforce

**DOI:** 10.3389/fgene.2023.1337366

**Published:** 2024-01-08

**Authors:** Maria C. Katapodi, Carla Pedrazzani, Sivia Barnoy, Efrat Dagan, Muriel Fluri, Tarsha Jones, Sue Kim, Meghan L. Underhill-Blazey, Melissa K. Uveges, Andrew A. Dwyer

**Affiliations:** ^1^ International Nursing CASCADE Consortium, Basel, Switzerland; ^2^ Department of Clinical Research, University of Basel, Basel, Switzerland; ^3^ Department of Business Economics, Health and Social Care, University of Applied Sciences and Arts of Southern Switzerland, Manno, Switzerland; ^4^ Nursing Department, Tel-Aviv University, Tel Aviv, Israel; ^5^ The Cheryl Spencer Department of Nursing, University of Haifa, Haifa, Israel; ^6^ Inselspital, Bern University Hospital, Bern, Switzerland; ^7^ Christine E. Lynn College of Nursing, Florida Atlantic University, Boca Raton, FL, United States; ^8^ College of Nursing, Yonsei University, Seoul, Republic of Korea; ^9^ School of Nursing, University of Rochester, Rochester, NY, United States; ^10^ William F. Connell School of Nursing, Boston College, Boston, MA, United States; ^11^ Massachusetts General Hospital, Harvard Center for Reproductive Medicine, Boston, MA, United States

**Keywords:** international perspective, nursing practice, interprofessional collaboration, nursing code of ethics, advocacy, family communication and coping, cascade genetic testing, surveillance

## Abstract

**Introduction:** Efforts are needed across disciplines to close disparities in genomic healthcare. Nurses are the most numerous trained healthcare professionals worldwide and can play a key role in addressing disparities across the continuum of care. ACCESS is an empirically-based theoretical framework to guide clinical practice in order to ameliorate genomic disparities.

**Methods:** The framework was developed by the International Nursing CASCADE Consortium based on evidence collected between 2005 and 2023 from individuals and families of various ethnic backgrounds, with diverse hereditary conditions, and in different healthcare systems, i.e., Israel, Korea, Switzerland, and several U.S. States. The components of the framework were validated against published scientific literature.

**Results:** ACCESS stands for Advocating, Coping, Communication, cascadE Screening, and Surveillance. Each component is demonstrated in concrete examples of clinical practice within the scope of the nursing profession related to genomic healthcare. Key outcomes include advocacy, active coping, intrafamilial communication, cascade screening, and lifelong surveillance. Advocacy entails timely identification of at-risk individuals, facilitating referrals to specialized services, and informed decision-making for testing. Active coping enhances lifelong adaptation and management of disease risk. Effective intrafamilial communication of predisposition to hereditary disease supports cascade testing of unaffected at-risk relatives. Lifelong surveillance is essential for identifying recurrence, changes in health status, and disease trajectory for life-threatening and for life-altering conditions.

**Discussion:** ACCESS provides a standardized, systematic, situational, and unifying guide to practice and is applicable for nursing and for other healthcare professions. When appropriately enacted it will contribute towards equitable access to genomic resources and services.

## 1 Introduction

While the “genomic era” introduced a new understanding of health and illness, it is paralleled by significant disparities in accessing genomic services and benefiting from technological advances, raising concerns about growing disparities in healthcare (National Academies of Sciences Engineering and Medicine, 2018). Genomic disparities affect patients, at-risk individuals, and families and are particularly prominent for racial, ethnic, and gender minorities, and for children, medically underserved, and geographically dispersed groups. Barriers to genomic healthcare are multilevel and include health finance structures, societal and cultural norms, provider bias, and concerns of discrimination and misuse of genomic information. An important contributor to genomic disparities is the relative lack of genetic specialists (Baars M et al., 2005; Ormond et al., 2018).

Nurses are the most numerous and among the most trusted of health professionals with a global workforce of 27.9 million (World Health Organization, 2020) and provide services to various settings, from remote rural areas to highly specialized centers. Nurses can play an important role in genomic healthcare after integrating genomic competences in nursing practice (Calzone et al., 2010). However, there is a need for a unifying model to guide nursing practice and surmount the growing genomic health disparities. To fill this gap, this Perspective presents ACCESS, an empirically-based theoretical framework that was developed by a panel of nurses from different countries and healthcare systems, studying different populations and genomic conditions.

## 2 Methods

ACCESS is based on a structured, rigorous process synthesizing empirical evidence with diverse populations in terms of gender, race and ethnicity, geography, and healthcare systems. It utilizes findings from more than sixty peer-reviewed publications of the investigators involved in the development of the framework over the past 18 years (2005–2023) on “common”, life-threatening conditions, e.g., hereditary breast and ovarian cancer (HBOC), and on rare, life-altering conditions, e.g., Kallmann syndrome (Supplementary Table S1). The development of ACCESS involved a four-step sequential process, i.e., reflection, mapping, refinement, and validation, with iterative review and discussion at each step. The framework is based on the critical assumption that nursing employs a person- and family-centered approach to care. First, investigators identified salient findings from their published work and reflected on broad themes running through studies to identify desired “key” outcomes for reducing disparities and improving genomic healthcare. Second, identified outcomes were mapped across populations, countries, and healthcare systems to chart similarities and coherence. Third, themes were organized according to the continuum of care, from primary prevention to rehabilitation. Last, as a validation step, and a safeguard against potential bias, we juxtaposed our findings against studies focusing on genomic disparities that were identified by a systematic scoping review and a health policy analysis that examined the current state of genomics in nursing (Puddester et al., 2023; Thomas et al., 2023).

## 3 Results

**FIGURE 1:**
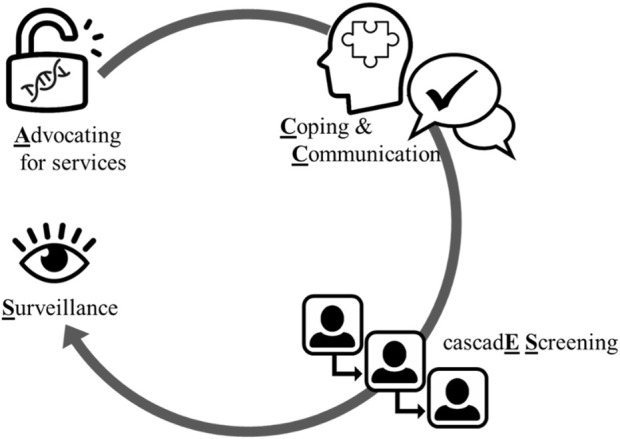
Schematic of the ACCESS framework. ACCESS proceeds from advocating for equitable access to care, to providing decisional support, to supporting active coping that precedes intra-familial communication of risk and cascade screening of relatives, and is followed by ongoing surveillance. Image credits: thenounproject.com.

ACCESS stands for Advocating, Coping, Communication, cascadE Screening, and Surveillance and provides a standardized, systematic, situational, and unifying guide to enable practicing nurses contribute towards decreasing disparities in genomic healthcare (Figure 1).

### 3.1 Advocating for access to services

Advocacy involves timely identification of at-risk individuals, facilitating access to reliable services, and promoting informed decision-making for testing as a prerequisite to decisions aligned with individual values and preferences. Advocating for access to services involves nurses, especially in primary care, taking a detailed medical history and a three-generation family history to identify at-risk individuals and refer them to specialized services. Addressing out-of-pocket costs and insurance barriers remove roadblocks to genomic services, especially for those who are less likely to have genetic testing due to financial barriers. In countries with national or mandatory insurance coverage (e.g., Switzerland, Korea, Israel), variations in insurance coverage may create disparities in accessing testing or potentially lifesaving risk-reducing surgeries (Barnoy et al., 2023).

Health literacy barriers among underserved, low income, and less educated communities hinders integration of genomic information in health decision-making. Nurses can reach individuals with limited health literacy and numeracy and increase access to genomic healthcare by using professional competencies in patient education and outreach, along with culturally and linguistically appropriate education materials. Effective strategies include limiting the amount of information delivered in one counseling session, using lay language and understandable and actionable terms, assessing patient comprehension, employing “teach-back” strategies, and employing digital health technologies (Barr et al., 2018).

### 3.2 Active coping and family communication

A family-based approach to communicating risk for genomic diseases can leverage bonds within a family network and can reach individuals with irregular interactions with healthcare providers. However, it is not uncommon for individuals with disease-causing variants to conceal genomic information from first-degree relatives as well as from more distant or estranged relatives (Srinivasan et al., 2020). Family communication involves navigating and managing complex and potentially conflicting individual and family needs. The process is most effective when individuals engage in active coping strategies (e.g., seek expert advice and support) as opposed to avoidant coping. Active coping precedes management of disease risk, while intra-familial communication is essential for subsequent cascade testing of relatives. This is especially important under the current regulatory milieu that precludes direct contact between healthcare providers and at-risk relatives without the consent of the tested individual (Henrikson et al., 2020). Individuals with disease-causing variants need support to initiate disclosure of testing results to relatives, and relatives’ active coping response will lead to seeking reliable information and support from healthcare professionals, and to an informed decision regarding initiating or forging cascade testing. Nurses can support and empower individuals with disease-causing variants by adopting a patient-centered, tailored approach that fosters therapeutic relationships and open dialogue, considering the realm of the individual, family, and healthcare system.

### 3.3 Cascade genetic screening

Using genetic testing to identify asymptomatic individuals with disease-causing pathogenic/likely pathogenic (P/LP) variants is an important genomic public health intervention. Cascade screening refers to the process of extending genomic services to biological relatives of individuals harboring disease-causing P/LP variant(s) to inform risk management of relatives, while decreasing unnecessary healthcare expenditures for relatives that test negative. The U.S. Centers for Disease Control and Prevention, Office of Public Health Genomics classifies HBOC, Lynch syndrome (LS), and familial hypercholesterolemia (FH) as “Tier-1” genetic conditions (Khoury and Dotson, 2021). Tier 1 conditions are identified through genetic testing, and are actionable, meaning that implementing evidence-based guidelines can result in improved, measurable public health outcomes. Cascade screening involves asymptomatic individuals being educated about and considering testing for the P/LP variant in the family. Cascade screening enables asymptomatic individuals to access specialized services, receive accurate information, and initiate appropriate risk management.

Post-testing consultation usually includes a discussion about cascade screening yet, this aspect typically represents a relatively small portion of the patient encounter. Cascade screening may be more likely when healthcare providers have direct contact with relatives (Frey et al., 2022). However, approximately 70% of countries worldwide have in place legislation regarding privacy and protection of personal information, including genomic information (United Nations Conference on Trade and Development, 2021). Such legislation precludes healthcare providers from directly contacting at-risk relatives. Nurse-led, pre- and/or post-genetic testing consultations focused on enhancing active coping and family communication can also facilitate disclosure of genomic information and catalyze cascade genetic screening. A nurse-led cascade screening program for FH in Western Australia demonstrated cost-effectiveness and reduced incident of cardiovascular disease by 25%–50% over 10 years (Ademi et al., 2014). A cascade genetic screening program for FH in the Netherlands, facilitated by specialized nurses who carried out home visits for consent, pre-testing counseling, blood sampling for genetic testing, and collection of personal and family data, yielded a participation rate of 90% within the first 5 years, and identified approximately 3% of the FH population in the Netherlands (Umans-Eckenhausen et al., 2001). Within 20 years, the program has identified and treated an estimated 42% of the total FH population in the Netherlands (Besseling et al., 2015).

### 3.4 Ongoing surveillance

After receiving a genomic diagnosis, individuals face multiple health- and life-altering decisions that relate to risk-reducing and screening behaviors, reproduction, interpersonal relationships, occupation, and career. Continuity of care and long-term patient-provider relationships are the basis for assessing psychosocial adaptation to living with a genetic diagnosis. For individuals harboring P/LP variants in genes underlying life-threatening conditions (e.g., HBOC), ongoing surveillance with biomarkers and serial imaging is critical for managing risk and detecting cancer (re)occurrence. For rare, non-life-threatening diseases (e.g., Kallmann syndrome), ongoing surveillance is essential for monitoring disease progression and for comprehensive chronic care. The therapeutic relationship that grows from continuity of care helps identify patients’ challenges and creates opportunities to intervene with education and counseling or appropriate referrals (e.g., reproductive specialists), thus, supporting comprehensive, coordinated, inter-professional care.

### 3.5 Applying the ACCESS framework to nursing practice

ACCESS provides a standardized, systematic, situational, and unifying guide to nursing practice that enables practicing nurses to help close disparities in genomic healthcare. ACCESS embeds genomics in already established professional nursing roles, which when appropriately enacted, enable equitable access to genomic resources and services. Table 1 provides concrete examples of nursing practice relating to each of the components.

**TABLE 1:** Examples of applying the ACCESS framework to nursing practice.

“A” *advocacy*
• Enhance access: provide documentation to facilitate insurance coverage for genetic counselling and testing and to address other economic barriers, e.g., coverage for subsequent treatment
• Decisional support: use active listening techniques to reflect back values and preferences for genetic testing decisions
• Genetic literacy and numeracy: elicit and evaluate understanding of and attitudes towards genomic healthcare with tailored and linguistically appropriate education materials
• Identification: identify “red flags” indicating a genomic condition in personal or family health history. Apply the “too”/“two” rule, i.e., recognizing that genomic conditions may produce extreme phenotypes (“too”) or may cause disease in bilateral organs (“two”). Taking and documenting a 3-generation family history using standard nomenclature; identifying those who could benefit from genomic services
• Referrals: Provide information and anticipatory guidance about genetic counselling and make referrals to such services
“C” *Coping*
• Addressing unique needs of caregivers: evaluate levels of distress and cancer worry and develop supportive care resources
• Individualized approach: tailor approach to respond to client’s priority concerns and informational needs. Use “teach back” to assess and ensure comprehension
• Narrative nudges:highlight aspects of patient narratives that shift the perspective towards “living with” a diagnosis rather than being “defined by” a diagnosis
• Reframing, emotional support, and stress reducing interventions: use active listening and therapeutic communication to reframe fears and concerns as opportunities to improve health and support relatives, organize personal exchanges with other affected persons
• Therapeutic listening: use a strengths-based approach to foster confidence in coping with challenging situations and health threats
• Uncertainty management: assess for sources of and responses to uncertainty, offer psychosocial and educational support
*“C” Communication of risk*
• Coaching: provide tailored coaching with modeling and opportunities to build self-efficacy
• Cultural norms: assess cultural norms and patterns of familial communication
• Supporting and empowering: Inquire about people who can initiate and maintain family communication about hereditary conditions. Support in informing biological relatives with letters and build communication strategies for direct information
• Therapeutic education: provide information, supporting documents, anticipatory guidance on possible emotional reactions, and reinforcement to build self-efficacy for family discussions
“ES” *CascadE Screening*
• Nurse-led interventions: implement rigorous evidence-based interventions that enhance uptake of cascade genetic screening among relatives
• Referral sources: provide information on costs of genetic counselling and testing, and insurance coverage to relatives
• Resource materials: have materials and resources at hand regarding disease management, prophylaxis, expert care that can be passed on to relatives
“S” *Surveillance*
• Continuity and long-term care: assist navigating through lifelong challenges and life-altering decisions (i.e., risk-reducing surgery, fertility preservation). Provide long-term support in specialized clinics
• Disease recurrence: follow established, evidence-based ongoing disease-specific surveillance activities (i.e., imaging, blood tests, biomarkers, etc.)
• Lifestyle, stress reduction, and health promoting behavioral counseling: enhance health promoting and risk-reducing behaviors. Recognize and address patient experience about risk-reducing surgery, support living with side effects
• Therapeutic relationship: use trust in the therapeutic relationship to provide ongoing coping reinforcement, emotional support, and strengths-based encouragement tailored to the individual, familial, and cultural norms
• Referrals for additional services: assess for changing needs and refer for additional services (e.g., psychologists, reproductive specialists, etc.), providing comprehensive, coordinated and inter-professional care

## 4 Discussion

Evidence of growing genomic health disparities and implications for individuals, families, and communities present an urgent call for action. A number of barriers must be overcome to ameliorate genomic disparities and harness the full potential of genomics for improving prevention, screening, diagnosis, and treatment for individuals, families, and communities. Nursing has a long history of promoting self-care and delivering holistic person-, family-, and community-centered care, that is built on sound assessment, effective communication, and therapeutic education (Fee and Bu, 2010). To keep pace with the growing integration of genomics into healthcare delivery, nurses at all levels of practice must apply relevant competencies in practice. Nurses are the most numerous of trained healthcare professionals (World Health Organization, 2020), involved in interprofessional care delivered in ambulatory and community-based facilities, hospitals, and integrated healthcare systems. Nurses provide care in a variety of settings ranging from remote, rural, and medically underserved communities to urban and tertiary care settings. Most importantly, nurses and nursing practice worldwide are governed by the International Council of Nurses Code of Ethics, which is concerned with issues of privacy, confidentiality, advocacy, equity, and responsibility (International Council of Nurses, 2021). Specifically, article 1.3 clarifies that nurses ensure that the individual and family receive understandable, accurate, sufficient and timely information on which to base decisions for care and treatment. Articles 1.4 and 1.5 hold nurses accountable towards confidentiality of personal information and respect for privacy of individuals needing care. Specifically, article 2.9 discusses explicitly the role of nursing in safeguarding privacy and autonomy of decision-making regarding genomic information and fostering access to genomic technologies. Finally, articles 1.6, 1.7, 4.5, and 4.7 hold nurses responsible for initiating and supporting actions that meet the health and social needs of all people, collaborate with other disciplines to uncover social determinants of health, and advocate and promote equity and social justice in accessing healthcare and other social and economic services. As such, nurses worldwide are uniquely positioned to play a key role in bridging disparities in genomic healthcare. Nursing actions include safeguarding individual rights to privacy and confidentiality, advocating for equitable access to genomic services, as well as monitoring and calling out health practices and policies that contribute to widening healthcare disparities or to discriminatory practices related to genomic information.

We posit that ACCESS is a novel, simple, yet, practical framework that can be part of a multi-level approach to increase integration of genomic care into nursing practice. Importantly, the framework is not disease-specific, but rather it is flexible and relevant for a broad range of conditions and healthcare systems. Although ACCESS was initiated by and builds on work of an international nursing consortium, it is applicable to other disciplines involved in genomic healthcare, ranging from direct care provision at the bedside to health policy. The universal shortage of genomic specialists requires that healthcare providers and policymakers seek for novel and sustainable solutions regarding widespread implementation of germline testing. This is in addition to the need for streamlining educational efforts regarding implications of genetic testing, especially for prevention and targeted therapeutics (Al-Sukhun et al., 2023; Brown et al., 2023; Moyo et al., 2023). Implementation of a systematic guide like the ACCESS framework, and partnering with the nursing workforce who is a major stakeholder in promoting health equity, may facilitate initiatives such as the Rare Genomes Project (RGP) (Serrano et al., 2023) and the Genomic Answers for Kids (GA4K) (Kane et al., 2023) reduce barriers and inequalities for underrepresented patients with rare genomic disorders and for children, respectively. Disparities are a global concern of patients and families, communities, providers, health systems, and public health agencies and are among the most anticipated challenges for healthcare policy for the next decade (Dolan et al., 2023; Hull et al., 2023; Phillips et al., 2023). Ameliorating healthcare disparities, including genomic disparities, requires a unifying, comprehensive, and multilevel approach that can be embraced by and enacted upon across disciplines (e.g., bioethics, genetic counseling, medical genetics, medicine, nursing, social work, etc.). The components of ACCESS can be integrated both into education and practice as a standardized and systematic guide that can help create and maintain a pipeline of trained healthcare professionals who are vigilant about genomic disparities and engage in actions that equitably improve health and wellbeing for patients, families, and communities.

One potential limitation is that the ACCESS framework is that it is based on empirical evidence from studies conducted by the members of our consortium and not on a systematic literature search. Although members of our consortium conducted their studies worldwide, in a variety of settings, and with diverse patient populations, we cannot preclude the possibility of bias. However, the components of the ACCESS framework are consistent with conclusions of a recently published scoping review that examined health disparities and the current state of genomics in nursing (Thomas et al., 2023) and with other primary studies and systematic reviews referenced in this Perspective. Nevertheless, we propose that future studies should focus on implementation of the framework and evaluation of its effectiveness with rigorous research designs.

## Data Availability

The original contributions presented in the study are included in the article/Supplementary Material, further inquiries can be directed to the corresponding author.
