# Supplementary material for: ACCESS: an empirically-based framework developed by the International Nursing CASCADE Consortium to address genomic disparities through the nursing workforce
Source: Front Genet. 2024 Jan 8;14:1337366. doi: 10.3389/fgene.2023.1337366 (PMC10804848; doi:10.3389/fgene.2023.1337366)
Supplement: Supplementary file 1 [file Table1.docx]

Supplementary Material

ACCESS: an empirically-based framework developed by the International Nursing CASCADE Consortium to address genomic disparities through the nursing workforce

Maria C. KATAPODI*, Carla PEDRAZZANI, Sivia BARNOY, Efrat DAGAN, Muriel FLURI, Tarsha JONES, Sue KIM, Meghan L. UNDERHILL-BLAZEY, Melissa K. UVEGES, Andrew A. DWYER*.

*** Correspondence:**

Maria C. Katapodi, PhD, RN, FAAN - [maria.katapodi@unibas.ch](mailto:maria.katapodi@unibas.ch)

Andrew A. Dwyer, PhD, FNP-BC, FNAP, FAAN - [andrew.dwyer@bc.edu](mailto:andrew.dwyer@bc.edu)

# Supplementary Table

**Table 1. Identified themes for the ACCESS framework based on similarities and differences between common, life-threatening diseases and rare, life-altering conditions.**

|  |  | Supporting studies | |
| --- | --- | --- | --- |
| Common theme | **Nursing care elements** | **Common, life-threatening diseases** | **Rare, life-altering diseases** |
| Advocacy | - Enhance access (supporting reimbursement and access to insurance) - Decisional support - Genetic literacy - Identification (3-generation family history) - Referrals for genetic services | Chittenden et al., 2021  Dagan et al., 2021  Dwyer et al., 2020; 2022a  Furniss et al., 2021  Jones et al., 2016; 2021a; 2021b; 2021c  Katapodi & Aouizerat, 2005  Katapodi et al., 2011a; 2013b; 2017a  Nikolaidis et al., 2019  Pedrazzani et al., 2021; 2022  Salikhanov et al., 2021  Underhill & Crotser, 2014  Underhill et al., 2017  Underhill-Blazey et al., 2019b; 2021 | Dwyer et al., 2014; 2019; 2022c  Dzemaili et al., 2017 |
| Active Coping | - Addressing unique needs of caregivers - Individualized approach - Narrative nudges - Reframing, emotional support and stress reducing interventions - Therapeutic listening - Uncertainty management | Dwyer et al., 2020; 2022b  Jones et al., 2021c  Katapodi et al., 2011b; 2013a; 2018  Underhill & Dickerson, 2011  Underhill et al., 2012; 2015; 2018  Underhill-Blazey et al., 2019a; 2022a; 2022b | Dwyer et al., 2014; 2019; 2022c  Dzemaili et al., 2017  Schwartz et al., 2022 |
| Intrafamilial Communication | - Culturally tailored and personalized coaching - Modeling and practice - Supporting and empowering disclosure - Therapeutic education | Aceti et al., 2022  Barnoy et al., 2023  Baroutsou et al., 2021  Dwyer et al., 2020; 2022b  Katapodi et al., 2018  Kim et al., 2021  Pedrazzani et al, 2022  Sarki et al., 2022  Simond et al, 2020 | Dwyer et al., 2014; 2019; 2022c  Dzemaili et al., 2017 |
| Cascade Screening | - Decisional support - Family dynamics and family variant testing - Referral sources - Resource materials | Dagan et al., 2017  Dwyer et al., 2020; 2022b  Gilbar et al., 2016  Katapodi et al., 2017b; 2020  Kim et al., 2021  Nikolaidis et al., 2018  Sarki et al., 2022 | Boehm et al., 2015  Dwyer et al., 2022c  Maione et al., 2018 |
| Lifelong Surveillance | - Continuity and long-term care - Disease recurrence (HBOC/Lynch syndrome) - Lifestyle, stress reduction and health promoting behavioral counseling - Reversal (CHH/KS) - Therapeutic relationship - Referrals for additional services | Fadda et al., 2020  Jabaley et al., 2020  Katapodi et al., 2020  Schweighoffer et al., 2022  Tsvitman et al., 2021  Underhill et al., 2018  Underhill-Blazey et al., 2022a  Young et al., 2022 | Boehm et al., 2015  Dwyer et al., 2016 |

**References**

Aceti, M., Caiata-Zufferey, M., Schweighoffer, R., Baroutsou, V., Pedrazzani, C., Katapodi, M., Kim, S. (2022, June 1– 3). Is a gene running in your family? Perceptions of responsibility to inform relatives about genetic cancer predisposition and risk management: an in-depth qualitative Swiss-Korean comparative analysis [Conference session]. The 5th ELSI Congress: Innovating for a Just and Equitable Future, New York.

Barnoy, S., Dagan, E., Kim, S., Caiata-Zufferey, M., Katapodi, M.C., the CASCADE and the K-CASCADE Consortia. (2023). Privacy and utility of genetic testing in families with hereditary cancer syndromes living in three countries: The International Cascade Genetic Screening experience. Frontiers in Genetics. 14, 1109431. doi: 10.3389/fgene.2023.1109431.

Baroutsou, V., Underhill-Blazey, M.L., Appenzeller-Herzog, C., Katapodi, M.C. (2021). Interventions Facilitating Family Communication of Genetic Testing Results and Cascade Screening in Hereditary Breast/Ovarian Cancer or Lynch Syndrome: A Systematic Review and Meta-Analysis. Cancers, 13(4):925. doi: 10.3390/cancers13040925.

Caiata-Zufferey, M., Pagani, O., Cina, V., Membrez, V., Taborelli, M., Unger, S., Murphy, A., Monnerat, C., Chappuis, P.O. (2015). Challenges in managing genetic cancer risk: a long-term qualitative study of unaffected women carrying BRCA1/BRCA2 mutations. Genetics in Medicine, 17(9):726-32. doi: 10.1038/gim.2014.183.

Chittenden, A., Haraldsdottir, S., Ukaegbu, C., Underhill-Blazey, M., Gaonkar, S., Uno, H., Brais, L.K., Perez, K., Wolpin, B.M., Syngal, S., Yurgelun, M.B. (2021). Implementing Systematic Genetic Counseling and Multigene Germline Testing for Individuals With Pancreatic Cancer. JCO Oncology Practice, 17(2):e236-e247. doi: 10.1200/OP.20.00678.

Dagan, E., Amit, Y., Sokolov, L., Litvak, P., Barnoy, S. (2021). Integrating genetic professional skills into nursing practice: Results from a large cohort of Israeli nurses. Journal of Nursing Scholarship, 53(6):753-761. doi: 10.1111/jnu.12686.

Dagan, E., Friedman, E., Birenbaum-Carmeli, D., Feldman,‎ B. (2017). Performing and Declining PGD: Accounts of Jewish Israeli Women Who Carry a BRCA1/2 Mutation or Partners of Male Mutation Carriers. Journal of Genetic Counseling. 26:1070-1079. doi: 10.1007/s10897-017-0087-6.

Dwyer, A.A. (2020). Psychosexual effects resulting from delayed, incomplete, or absent puberty. Current Opinion in Endocrine and Metabolic Research, 14:15-21. doi: 10.1016/j.coemr.2020.04.003.

Dwyer, A.A., Calzone, K.A., Dewell, S., Badzek, L., Patch, C. (2022a). Correspondence on "Ensuring best practice in genomics education and evaluation: Reporting item standards for education and its evaluation in genomics (RISE2 Genomics)" by Nisselle et al. Genetics in Medicine, 24(4):962-963. doi: 10.1016/j.gim.2021.11.023.

Dwyer, A.A., Hesse-Biber, S., Flynn, B., Remick, S. (2020). Parent of Origin Effects on Family Communication of Risk in BRCA+ Women: A Qualitative Investigation of Human Factors in Cascade Screening. Cancers, 12(8):2316. doi: 10.3390/cancers12082316.

Dwyer, A.A., Hesse-Biber, S., Shea, H., Zeng, Z., Yi S. (2022b). Coping response and family communication of cancer risk in men harboring a BRCA mutation: A mixed methods study. Psychooncology, 31(3):486-495. doi: 10.1002/pon.5831.

Dwyer, A.A., Quinton, R., Morin, D., Pitteloud, N. (2014). Identifying the unmet health needs of patients with congenital hypogonadotropic hypogonadism using a web-based needs assessment: implications for online interventions and peer-to-peer support. Orphanet Journal of Rare Diseases, 11;9:83. doi: 10.1186/1750-1172-9-83.

Dwyer, A.A., Raivio, T., Pitteloud, N. (2016). MANAGEMENT OF ENDOCRINE DISEASE: Reversible hypogonadotropic hypogonadism. European Journal of Endocrinology, 174(6):R267-74. doi: 10.1530/EJE-15-1033.

Dwyer, A.A., Shen, H., Zeng, Z., Gregas, M, Zhao, M. (2021). Framing Effects on Decision-Making for Diagnostic Genetic Testing: Results from a Randomized Trial. Genes, 12(6):941. doi: 10.3390/genes12060941.

Dwyer, A.A., Smith, N., Quinton, R. (2019). Psychological Aspects of Congenital Hypogonadotropic Hypogonadism. Frontiers in Endocrinology, 10:353. doi: 10.3389/fendo.2019.00353.

Dwyer, A.A., Uveges, M.K., Dockray, S., Smith, N. (2022c). Exploring Rare Disease Patient Attitudes and Beliefs regarding Genetic Testing: Implications for Person-Centered Care. Journal of Precision Medicine, 12(3):477. doi: 10.3390/jpm12030477.

Dzemaili, S., Tiemensma, J., Quinton, R., Pitteloud, N., Morin, D., Dwyer, A.A. (2017). Beyond hormone replacement: quality of life in women with congenital hypogonadotropic hypogonadism. Endocrine Connections, 6(6):404-412. doi: 10.1530/EC-17-0095.

Fadda, M., Chappuis, P.O., Katapodi, M.C., Pagani, O., Monnerat, C., Membrez, V., Unger, S., Caiata Zufferey, M. (2020). Physicians communicating with women at genetic risk of breast and ovarian cancer: Are we in the middle of the ford between contradictory messages and unshared decision making? PLoS One, 15(10):e0240054. doi: 10.1371/journal.pone.0240054.

Furniss, C.S., Yurgelun, M.B., Ukaegbu, C., Constantinou, P.E., Lafferty, C.C., Talcove-Berko, E.R., Schwartz, A.N., Stopfer, J.E., Underhill-Blazey, M., Kenner, B., Nelson, S.H. (2021). Novel Models of Genetic Education and Testing for Pancreatic Cancer Interception: Preliminary Results from the GENERATE Study. Cancer Prevention Research, 14(11), pp.1021-1032. doi: 10.1158/1940-6207.CAPR-20-0642.

Gilbar, R., Barnoy, S. (2012). Disclosure of genetic information to relatives in Israel: between privacy and familial responsibility. New Genetics and Society, 31(4), 391-407.‏ doi: 10.1080/14636778.2012.687135.

Gilbar, R., Shalev, S., Spiegel, R., Pras, E., Berkenstadt, M., Sagi, M., Ben-Yehuda, A., Mor, P., Perry, S., Zaccai, T.F., Borochowitz, Z., Barnoy, S. (2016). Patients’ attitudes towards disclosure of genetic test results to family members: the impact of patients’ sociodemographic background and counseling experience. Journal of Genetic Counseling, 25(2), 314-324.‏ doi: 10.1007/s10897-015-9873-1.

Jabaley, T., Underhill-Blazey, M.L., Berry, D.L. (2020). Development and Testing of a Decision Aid for Unaffected Women with a BRCA1 or BRCA2 Mutation. Journal of Cancer Education, 35(2):339-344. doi: 10.1007/s13187-019-1470-9.

Jones, T, Guzman, A., Silverman, T., Freeman, K., Kukafka, R., Crew, K. (2021a). Perceptions of Racially and Ethnically Diverse Women at High Risk of Breast Cancer Regarding the Use of a Web-Based Decision Aid for Chemoprevention: Qualitative Study Nested Within a Randomized Controlled Trial. Journal of Medical Internet Research; 23(6):e23839. doi: 10.2196/23839.

Jones, T., Howard, H., Freeman-Costin, K., Creighton, A., Wisdom-Chamber, K., Underhill-Blazey, M. (2021b). Knowledge and perceptions of BRCA1/2 genetic testing and needs of diverse women with a personal or family history of breast cancer in South Florida. Journal of Community Genetics, 12(3):415-429. doi: 10.1007/s12687-021-00507-6.

Jones, T., Lockhart, J.S., Mendelsohn-Victor, K.E., Duquette, D., Northouse, L.L., Duffy, S.A., Donley, R., Merajver, S.D., Milliron, K.J., Roberts, J.S., Katapodi, M.C. (2016). Use of Cancer Genetics Services in African-American Young Breast Cancer Survivors. American Journal of Preventive Medicine, 51(4):427-36. doi: 10.1016/j.amepre.2016.03.016.

Jones, T., McCarthy, A.M., Kim, Y., Armstrong K. (2017). Predictors of BRCA1/2 genetic testing among Black women with breast cancer: a population-based study. Cancer Medicine, 6(7):1787-1798. doi: 10.1002/cam4.1120.

Jones, T., Trivedi, M.S., Jiang, X., Silverman, T., Underhill, M., Chung, W.K., Kukafka, R., Crew, K.D. (2021c). Racial and Ethnic Differences in BRCA1/2 and Multigene Panel Testing Among Young Breast Cancer Patients. Journal of Cancer Education, 36(3):463-469. doi: 10.1007/s13187-019-01646-8.

Katapodi, M.C., Aouizerat, B.E. (2005). Do women in the community recognize hereditary and sporadic breast cancer risk factors? Oncology Nursing Forum, 32(3):617-23. doi: 10.1188/05.ONF.617-623.

Katapodi, M.C., Duquette, D., Yang, J.J., Mendelsohn-Victor, K., Anderson, B., Nikolaidis, C., Mancewicz, E., Northouse, L.L., Duffy, S., Ronis, D., Milliron, K.J., Probst-Herbst, N., Merajver, S.D., Janz, N.K., Copeland, G., Roberts, S. (2017a). Recruiting families at risk for hereditary breast and ovarian cancer from a statewide cancer registry: a methodological study. Cancer Causes and Control. 28(3):191-201. doi: 10.1007/s10552-017-0858-2.

Katapodi, M.C., Jung, M., Schafenacker, A.M., Milliron, K.J., Mendelsohn-Victor, K.E., Merajver, S.D., Northouse, L.L. (2018). Development of a Web-based Family Intervention for BRCA Carriers and Their Biological Relatives: Acceptability, Feasibility, and Usability Study. JMIR Cancer, 13;4(1):e7. doi: 10.2196/cancer.9210.

Katapodi, M.C., Ming, C., Northouse, L.L., Duffy, S.A., Duquette, D., Mendelsohn-Victor, K.E., Milliron, K.J., Merajver, S.D., Dinov, I.D., Janz, N.K. (2020). Genetic Testing and Surveillance of Young Breast Cancer Survivors and Blood Relatives: A Cluster Randomized Trial. Cancers, 12(9):2526. doi: 10.3390/cancers12092526.

Katapodi, M.C., Munro, M.L., Pierce, P.F., Williams, R.A. (2011a). Psychometric testing of the decisional conflict scale: genetic testing hereditary breast and ovarian cancer. Nursing Research, 60(6):368-77. doi: 10.1097/NNR.0b013e3182337dad.

Katapodi, M.C., Northouse, L.L., Milliron, K.J., Liu, G. Merajver, S.D. (2013a). Individual and family characteristics associated with BRCA1/2 testing in high-risk families. Psycho-Oncology, 22, 1336 – 1343, doi: 10.1002/pon.3139.

Katapodi, M.C., Northouse, L.L., Pierce, P.F., Milliron, K.J., Liu, G., Merajver, S.D. (2011b). Differences between women who pursued genetic testing for HBOC and their at-risk relatives who did not. Oncology Nursing Forum, 38(5), 572 – 581, doi: 10.1188/11.ONF.572-581.

Katapodi, M.C., Northouse, L.L., Schafenacker, A.M., Duquette, D., Duffy, S.A., Ronis, D.L., Anderson, B., Janz, N.K., McLosky, J., Milliron, K.J., Merajver, S.D., Duong, L.M., Copeland, G. (2013b). Using a state cancer registry to recruit young breast cancer survivors and high-risk relatives: protocol of a randomized trial testing the efficacy of a targeted versus a tailored intervention to increase breast cancer screening. BMC Cancer, 13(97), doi:10.1186/147124071397.

Katapodi, M.C., Viassolo, V., Caiata-Zufferey, M., Nikolaidis, C., Bührer-Landolt, R., Buerki, N., Graffeo, R., Horváth, H.C., Kurzeder, C., Rabaglio, M., Scharfe, M., Urech, C., Erlanger, T.E., Probst-Hensch, N., Heinimann, K., Heinzelmann-Schwarz, V., Pagani, O., Chappuis, P.O. (2017b). Cancer Predisposition Cascade Screening for Hereditary Breast/Ovarian Cancer and Lynch Syndromes in Switzerland: Study Protocol. JMIR ResearchPprotocols, 6(9):e184. doi: 10.2196/resprot.8138.

Kim, S., Aceti, M., Baroutsou, V., Bürki, N., Caiata-Zufferey, M., Cattaneo, M., Chappuis, P.O., Ciorba, F.M., Graffeo-Galbiati, R., Heinzelmann-Schwarz, V., Jeong, J., Jung, M.M., Kim, S.W., Kim, J., Lim, M.C., Ming, C., Monnerat, C., Park, H.S., Park, S.H., …. Katapodi, M.C. (2021). Using a Tailored Digital Health Intervention for Family Communication and Cascade Genetic Testing in Swiss and Korean Families With Hereditary Breast and Ovarian Cancer: Protocol for the DIALOGUE Study. JMIR Research Protocols, 10(6):e26264. doi: 10.2196/26264.

Maione, L., Dwyer, A.A., Francou, B., Guiochon-Mantel, A., Binart, N., Bouligand, J., Young, J. (2018). GENETICS IN ENDOCRINOLOGY: Genetic counseling for congenital hypogonadotropic hypogonadism and Kallmann syndrome: new challenges in the era of oligogenism and next-generation sequencing. European Journal of Endocrinology, 178(3):R55-R80. doi: 10.1530/EJE-17-0749.

Nikolaidis, C., Duquette, D., Mendelsohn-Victor, K.E., Anderson, B., Copeland, G., Milliron, K.J., Merajver, S.D., Janz, N.K., Northouse, L.L., Duffy, S.A., Katapodi, M.C. (2019). Disparities in genetic services utilization in a random sample of young breast cancer survivors. Genetics in Medicine, 21(6):1363-1370. doi: 10.1038/s41436-018-0349-1.

Nikolaidis, C., Ming, C., Pedrazzani, C., van der Horst, T., Kaiser-Grolimund, A., Ademi, Z., Bührer-Landolt, R., Bürki, N., Caiata-Zufferey, M., Champion, V., Chappuis, P.O., Kohler, C., Erlanger, T.E., Graffeo, R., Hampel, H., Heinimann, K., Heinzelmann-Schwarz, V., Kurzeder, C., Monnerat, C., … Katapodi MC; for the CASCADE Consortium. (2018). Challenges and Opportunities for Cancer Predisposition Cascade Screening for Hereditary Breast and Ovarian

Park, S.Y., Kim, Y.L., Kim. S. (2020). Factors associated with the decision to undergo risk-reducing salpingo-oophorectomy among women at high risk for hereditary breast and ovarian cancer: a systematic review. Korean Journal of Women Health Nursing, 26(4): 285-299. https://doi.org/10.4069/kjwhn.2020.11.19

Pedrazzani, C., Aceti, M., Schweighoffer, R., Kaiser-Grolimund, A., Bürki, N., Chappuis, P.O., Graffeo, R., Monnerat, C., Pagani, O., Rabaglio, M., Katapodi, M.C., Caiata-Zufferey, M. (2022). The Communication Chain of Genetic Risk: Analyses of Narrative Data Exploring Proband–Provider and Proband–Family Communication in Hereditary Breast and Ovarian Cancer. Journal of Personalized Medicine, 12, 1249. https://doi.org/10.3390/jpm12081249

Pedrazzani, C., Ming, C., Bürki, N., Caiata-Zufferey, M., Chappuis, P.O., Duquette, D., Heinimann, K., Heinzelmann-Schwarz, V., Graffeo-Galbiati, R., Merajver, S.D., Milliron, K.J., Monnerat, C., Pagani, O., Rabaglio, M., Katapodi, M.C. (2021). Genetic Literacy and Communication of Genetic Information in Families Concerned with Hereditary Breast and Ovarian Cancer: A Cross-Study Comparison in Two Countries and within a Timeframe of More Than 10 Years. Cancers,13(24):6254. doi: 10.3390/cancers13246254.

Salikhanov, I., Heinimann, K., Chappuis, P., Buerki, N., Graffeo, R., Heinzelmann, V., Rabaglio, M., Taborelli, M., Wieser, S., Katapodi, M.C. (2021). Swiss cost-effectiveness analysis of universal screening for Lynch syndrome of patients with colorectal cancer followed by cascade genetic testing of relatives. Journal of Medical Genetics, jmedgenet-2021-108062. doi: 10.1136/jmedgenet-2021-108062.

Sarki, M., Ming, C., Aissaoui, S., Bürki, N., Caiata-Zufferey, M., Erlanger, T.E., Graffeo-Galbiati, R., Heinimann, K., Heinzelmann-Schwarz, V., Monnerat, C., Probst-Hensch, N., Rabaglio, M., Zürrer-Härdi, U., Chappuis, P.O., Katapodi, M.C., On Behalf Of The Cascade Consortium. (2022). Intention to Inform Relatives, Rates of Cascade Testing, and Preference for Patient-Mediated Communication in Families Concerned with Hereditary Breast and Ovarian Cancer and Lynch Syndrome: The Swiss CASCADE Cohort. Cancers, 14(7):1636. doi: 10.3390/cancers14071636.

Schwartz, T.S., Christensen, K.D., Uveges, M.K., Waisbren, S.E., McGuire, A.L., Pereira, S., Robinson, J.O., Beggs, A.H., Green, R.C., BabySeq Project Team, Bachmann, G.A., Rabson, A.B., Holm, I.A. (2022). Effects of participation in a U.S. trial of newborn genomic sequencing on parents at risk for depression. Journal of Genetic Counseling, 31(1):218-229. doi: 10.1002/jgc4.1475.

Schweighoffer, R., Aceti, M., Pedrazzani, C., Bürki, N., Chappuis, P.O., Graffeo Galbiati, R., Membrez, V., Monnerat, C., Pagani, O., Rabaglio, M., Unger, S., Katapodi, M.C., Caiata-Zufferey M. (2022, June 11-14). Making the right choice. How unaffected women carrying BRCA1/BRCA2 germline pathogenic variants decide for prophylactic mastectomy to reduce cancer risk [Conference session]. European Human Genetics Conference 2022, Vienna.

Shemesh, T., Barnoy, S. (2020). Assessment of the Intention to Use Mobile Health Applications Using a Technology Acceptance Model in an Israeli Adult Population. Telemedicine Journal and E-health, 26(9):1141-1149. doi: 10.1089/tmj.2019.0144.

Simond, E., Davoine, E., Katapodi, M.C., Unger S. (2020). Dissemination of Genetic Information in Swiss Families with Lynch Syndrome: A Qualitative Exploratory Study. Clinical Oncology & Research, 3(1): 2-5. doi:10.31487/j.COR.2020.01.01.

Tsvitman, I., Cohen Castel, O., Dagan E. (2021). Perceived patient-centered care is associated with reduced symptoms distress in cancer patients undergoing anti-cancer treatment. Supportive Care in Cancer, 1-9. doi: 10.1007/s00520-021-06200-1.

Underhill, M., Berry, D., Dalton, E., Schienda, J., Syngal, S. (2015). Patient experiences living with pancreatic cancer risk. Hereditary Cancer in Clinical Practice, 13(1):13. doi:10.1186/s13053-015-0034-1.

Underhill, M., Habin, K., Shannon, K. (2017). Perceptions of cancer risk, cause, and needs in participants from low socioeconomic background at risk for hereditary cancer. Behavioral Medicine, 43(4):259-67. doi: 10.1080/08964289.2016.1138925.

Underhill M, Hong F, Lawrence J, Blonquist T, Syngal S. (2018). Relationship between individual and family characteristics and psychosocial factors in persons with familial pancreatic cancer. Psychooncology, 27(7):1711-1718. doi: 10.1002/pon.4712.

Underhill, M.L., Crotser, C.B. (2014). Seeking balance: decision support needs of women without cancer and a deleterious BRCA1 or BRCA2 mutation. Journal of Genetic Counseling, 23(3):350-62. doi: 10.1007/s10897-013-9667-2.

Underhill, M.L., Dickerson, S.S. (2011). Engaging in medical vigilance: understanding the personal meaning of breast surveillance. Oncology Nursing Forum, 38(6):686-94. doi: 10.1188/11.ONF.686-694.

Underhill, M.L., Lally, R.M., Kiviniemi, M.T., Murekeyisoni, C., Dickerson, S.S. (2012). Living my family's story: identifying the lived experience in healthy women at risk for hereditary breast cancer. Cancer Nursing, 35(6):493-504. doi: 10.1097/NCC.0b013e31824530fa.

Underhill‐Blazey, M., Blonquist, T., Chittenden, A., Pozzar, R., Nayak, M., Lansang, K., Hong, F., Garber, J., Stopfer, J. E. (2021). Informing models of cancer genetics care in the era of multigene panel testing with patient‐led recommendations. Journal of Genetic Counseling, 30(1), 268-282. doi: 10.1002/jgc4.1317.

Underhill-Blazey, M., Blonquist, T., Lawrence, J., Hong, F., Yurgelun, M.B., Syngal, S. (2019a). Health behaviours and beliefs in individuals with familial pancreatic cancer. Familial Cancer, 18(4):457-464. doi: 10.1007/s10689-019-00143-7.

Underhill-Blazey, M., Rodriguez, D., Norton, S. A. (2022a). Scoping Review of Nonsurgical, Nonpharmacologic Interventions After Risk Reduction: Improving Quality of Life for Patients With Inherited Cancer Risk. Oncology Nursing Forum, 49 (3): 193-200. doi: 10.1188/22.ONF.193-200.

Underhill-Blazey, M., Stopfer, J., Chittenden, A., Nayak, M.M., Lansang, K., Lederman, R., Garber, J., Gundersen, D.A. (2019b). Development and testing of the KnowGene scale to assess general cancer genetic knowledge related to multigene panel testing. Patient Education and Counseling, 102(8):1558-1564. doi: 10.1016/j.pec.2019.04.014.

Underhill-Blazey, M. L., Zhang, Y., Stanek, S., Norton, S. (2022b). The Experience of Uncertainty in Individuals With High Risk for Pancreatic Cancer. Clinical Neuropharmacology, 10.1097/NCC.0000000000001171 doi: 10.1097/NCC.0000000000001171.

Young, J.M.L., Postula, K.J.V., Duquette, D., Gutierrez-Kapheim, M., Pan, V., Katapodi, M.C. (2022). Accuracy of Perceived Breast Cancer Risk in Black and White Women with an Elevated Risk. Ethnicity & Disease, 32(2):81-90. doi: 10.18865/ed.32.2.81.
